# Comparison of probabilistic tractography and tract-based spatial statistics for assessing optic radiation damage in patients with autoimmune inflammatory disorders of the central nervous system

**DOI:** 10.1016/j.nicl.2018.05.004

**Published:** 2018-05-08

**Authors:** Joseph Kuchling, Yael Backner, Frederike C. Oertel, Noa Raz, Judith Bellmann-Strobl, Klemens Ruprecht, Friedemann Paul, Netta Levin, Alexander U. Brandt, Michael Scheel

**Affiliations:** a Charité – Universitätsmedizin Berlin, corporate member of Freie Universität Berlin, Humboldt-Universität zu Berlin, and Berlin Institute of Health, Neurocure Cluster of Excellence, NeuroCure Clinical Research Center, NCRC Charité, Charitéplatz 1, 10117 Berlin, Germany; b Department of Neurology, Charité – Universitätsmedizin Berlin, Charitéplatz 1, 10117 Berlin, Germany; c Department of Neurology, The Agnes Ginges Center for Human Neurogenetics, Hadassah-Hebrew-University Medical Center, Kiryat Hadassah Ein kerem, Jerusalem 91120, Israel; d Experimental and Clinical Research Center, Max Delbrueck Center for Molecular Medicine and Charité – Universitätsmedizin Berlin, Charitéplatz 1, 10117 Berlin, Germany; e Department of Neurology, University of California, 1001 Health Sciences Road, Irvine Hall, Irvine, CA 92697, USA

**Keywords:** AD, axial diffusivity, AUC, area under the curve, CIS, clinically isolated syndrome, CON, Contrack, CSD, constrained spherical deconvolution, DTI, diffusion tensor imaging, DWI, diffusion weighted imaging, DW-MRI, diffusion weighted magnetic resonance imaging, FA, fractional anisotropy, FOD, fiber orientation distribution, HC, Healthy Control, JHU, Johns Hopkins University DTI white matter atlas, JUEL, Juelich histological atlas, LGN, lateral geniculate nucleus, MD, mean diffusivity, MS, multiple sclerosis, NMOSD, neuromyelitis optica spectrum disorder, OCT, optical coherence tomography, ON, optic neuritis, OR, optic radiation, PROB, probabilistic tractography, RD, radial diffusivity, RNFL, retinal nerve fiber layer thickness, ROC, receiver operating characteristic, ROI, region of interest, RRMS, relapsing-remitting multiple sclerosis, SD, standard deviation, SEM, standard error of the mean, TBSS, tract-based spatial statistics, DTI, Neuromyelitis optica, Multiple sclerosis, TBSS, Probabilistic tractography, Optic radiation

## Abstract

**Background:**

Diffusion Tensor Imaging (DTI) can evaluate microstructural tissue damage in the optic radiation (OR) of patients with clinically isolated syndrome (CIS), early relapsing-remitting multiple sclerosis and neuromyelitis optica spectrum disorders (NMOSD). Different post-processing techniques, e.g. tract-based spatial statistics (TBSS) and probabilistic tractography, exist to quantify this damage.

**Objective:**

To evaluate the capacity of TBSS-based atlas region-of-interest (ROI) combination with 1) posterior thalamic radiation ROIs from the Johns Hopkins University atlas (JHU-TBSS), 2) Juelich Probabilistic ROIs (JUEL-TBSS) and tractography methods using 3) ConTrack (CON-PROB) and 4) constrained spherical deconvolution tractography (CSD-PROB) to detect OR damage in patients with a) NMOSD with prior ON (NMOSD-ON), b) CIS and early RRMS patients with ON (CIS/RRMS-ON) and c) CIS and early RRMS patients without prior ON (CIS/RRMS-NON) against healthy controls (HCs).

**Methods:**

Twenty-three NMOSD-ON, 18 CIS/RRMS-ON, 21 CIS/RRMS-NON, and 26 HCs underwent 3 T MRI. DTI data analysis was carried out using JUEL-TBSS, JHU-TBSS, CON-PROB and CSD-PROB. Optical coherence tomography (OCT) and visual acuity testing was performed in the majority of patients and HCs.

**Results:**

Absolute OR fractional anisotropy (FA) values differed between all methods but showed good correlation and agreement in Bland-Altman analysis. OR FA values between NMOSD and HC differed throughout the methodologies (p-values ranging from p < 0.0001 to 0.0043). ROC-analysis and effect size estimation revealed higher AUCs and R^2^ for CSD-PROB (AUC = 0.812; R^2^ = 0.282) and JHU-TBSS (AUC = 0.756; R^2^ = 0.262), compared to CON-PROB (AUC = 0.742; R^2^ = 0.179) and JUEL-TBSS (AUC = 0.719; R^2^ = 0.161). Differences between CIS/RRMS-NON and HC were only observable in CSD-PROB (AUC = 0.796; R^2^ = 0.094). No significant differences between CIS/RRMS-ON and HC were detected by any of the methods.

**Conclusions:**

All DTI post-processing techniques facilitated the detection of OR damage in patient groups with severe microstructural OR degradation. The comparison of distinct disease groups by use of different methods may lead to different - either false-positive or false-negative - results. Since different DTI post-processing approaches seem to provide complementary information on OR damage, application of distinct methods may depend on the relevant research question.

## Highlights

• We describe a fully-automated optic radiation probabilistic tractography approach (CSD-PROB) that is feasible in healthy controls and patients with neuroimmunological diseases.
• We demonstrate CSD-PROB to yield superior sensitivity and specificity in differentiating NMOSD from healthy controls compared to TBSS-based optic radiation ROI methods and ConTrack-based probabilistic tractography.
• CSD-PROB shows significant differences between healthy controls and patients with clinically isolated syndrome and early multiple sclerosis without prior optic neuritis.
• The comparison of different subject groups by use of different DWI post-processing methods may lead to either false-positive or false-negative results, which may be of particular significance for future optic radiation DTI comparison in studies and meta-analyses.

## 1 Introduction

The optic radiation (OR) is an integral part of the afferent visual system and belongs to the most frequently affected white matter pathways in autoimmune neuroinflammatory disorders of the central nervous system, i.e. multiple sclerosis (MS) and neuromyelitis optica spectrum disorders (NMOSD) (Backner et al., 2018; Balcer et al., 2015; Bennett et al., 2015; Finke et al., 2018; Martínez-Lapiscina et al., 2014; Pache et al., 2016a, Pache et al., 2016b; Pache et al., 2016a, Pache et al., 2016b; Petzold et al., 2014; Pfueller and Paul, 2011; Scheel et al., 2014; Schmidt et al., 2017; Sinnecker et al., 2015b; Wingerchuk et al., 2015). Diffusion-weighted magnetic resonance imaging (DW-MRI) yields the potential to non-invasively investigate microstructural OR integrity (Assaf and Pasternak, 2008; Filippi et al., 2013).

A multitude of DW-MRI post-processing techniques have been used in recent studies to investigate OR damage in neuroinflammatory disorders (Hasan et al., 2011). TBSS is a widely used fully automated method to perform whole brain tract diffusion tensor imaging (DTI) analyses. ConTrack (CON-PROB) (Sherbondy et al., 2008a, Sherbondy et al., 2008b) and CSD-based probabilistic tractography (CSD-PROB) (Lim et al., 2015; Martínez-Heras et al., 2015; Tournier et al., 2007) provide high sensitivity to delineate tracts through crossing fiber regions (Auriat et al., 2015), facilitate the selection of pathways that connect two regions (Sherbondy et al., 2008b) and allow subsequent in-depth analysis, for example tract profiling, by calculating DTI values at different nodes along the OR. However, implementation of probabilistic tractography algorithms in the individual patient is frequently more time consuming due to manual predefinition of seed and target regions as well as manual or semi-automated cleaning of tractography results. Moreoever, accurate OR delineation in vivo is hampered by its complex structure with the sharp bending in the Meyer's loop (Martínez-Heras et al., 2015), the reduced fiber densitiy in this area compared to the body of the OR (Lim et al., 2015; Wu et al., 2012) and the presence of crossing fibers along the pathway (Sherbondy et al., 2008b).

Previous investigations using CON-PROB found OR DTI metrics to be altered in long-standing MS patients compared to healthy controls with correlations between OR FA and OR T2 lesion volume (Klistorner et al., 2014). A study investigating clinically isolated ON patients with CON-PROB found reduced fractional anisotropy (FA) and elevated radial diffusivity (RD) to be associated with OR lesions. No correlation between OR DTI and retinal nerve fiber layer thickness (RNFL) measured by optical coherence tomography (OCT) was found (Raz et al., 2015). By contrast, investigations using TBSS in MS patients with and without prior ON found strong correlations between RNFL and FA within the OR, suggesting trans-synaptic neurodegeneration after ON to explain the link between low RNFL thickness and low FA values in the OR (Scheel et al., 2014). These contradictory results fall in line with previous studies either favoring (Oertel et al., 2017; Pache et al., 2016a, Pache et al., 2016b; Reich et al., 2009; Rocca et al., 2013) or disfavoring (Dasenbrock et al., 2011) evidence on trans-neuronal changes in neuroinflammatory disorders. The conflicting diversity of published DTI studies might be partially owing to cohort inhomogeneities with regards to time from disease onset, severity of structural damage and clinical deficit as well as total and region-specific lesion load. Beyond this, the heterogeneous usage of different DTI post-processing techniques and their specific inherent limitations may account for inconsistent reports.

Validation studies of sensitivity, specificity and technical advantages and disadvantages of different DTI post-processing methods are thus highly required. Unfortunately, there is no “gold-standard” for non-invasive DTI-based OR tract-probing (Lim et al., 2015; Thomas et al., 2014), making comparability between methods and validation of techniques difficult. To overcome these limitations, different methods need to be compared against each other under one specific research question.

The purpose of our study was to compare distinct TBSS-based and probabilistic tractography-based approaches in the delineation of OR and the detection of OR damage. We therefore investigated OR damage with different severity levels and compared a) NMOSD patients with prior ON with suspected severe OR damage, b) clinically isolated syndrome (CIS) and early relapsing-remitting multiple sclerosis (RRMS) patients with ON and suspected moderate OR damage and c) CIS and early RRMS patients without prior ON and potential OR damage against healthy controls (HCs). We evaluated inter-method agreement of FA values and compared the capacity of all methods to detect OR FA differences in all patient cohorts compared to HCs.

## 2 Material and methods

### 2.1 Subjects

Sixty-two patients were retrospectively analyzed from our research database. This included CIS and early RRMS with ON (CIS/RRMS-ON), CIS and early RRMS without ON (CIS/RRMS-NON), NMOSD with ON (NMOSD-ON) as well as 26 HCs (see Table 1). All patients were examined under supervision of a board-certified neurologist at the NeuroCure Clinical Research Center, Charité-Universitätsmedizin Berlin between January 2011 and July 2015.

**Table 1:** Study cohort description.

	HC	CIS/RRMS-NON	CIS/RRMS-ON	NMOSD-ON
Subjects [n]	26	21	18	23
Sex [f(m)]	22(4)	11(10)	11(7)	20(3)
Age [years; mean ± SD]	43.7 ± 15.7	33.4 ± 8.6	31.2 ± 7.7	46.7 ± 14.5
Disease duration [months; mean ± SD]	*n.a.*	5.40 ± 6.67	4.63 ± 5.15	94.17 ± 95.72
EDSS [median; range]	*n.a.*	1.5 (0–4.0)	1.5 (0–3.5)	4.0 (0–6.5)
RRMS diagnosis [n]	*n.a.*	5 (23.8%)	3 (16.7%)	*n.a.*
AQP4-ab-positive [n]	*n.a.*	*n.a.*	*n.a.*	19
History of bilateral optic neuritis	*n.a.*	*n.a.*	0	4

HC = healthy control; CIS/RRMS-NON = clinically isolated syndrome without prior optic neuritis; CIS/RRMS-ON = clinically isolated syndrome with prior optic neuritis; NMOSD-ON = neuromyelitis optica spectrum disorder with prior optic neuritis; EDSS = expanded disability status scale; RRMS = relapsing-remitting multiple sclerosis; AQP4-ab-positive = Aquaporin-4-antibody positive.

We included 18 CIS/RRMS-ON patients from at the time of analysis 110 participants of the Berlin CIS Cohort study (ClinicalTrials.gov Identifier: NCT01371071; EA1/182/10). CIS/RRMS-ON patients were investigated following a first-time ON attack after 4.61 ± 5.51 months on average (range: 1–24 months) and showed no other neurological symptoms than ON-related visual dysfunction. All CIS/RRMS-ON patients presented with unilateral optic neuritis as their first clinical symptom. At the time of MRI examination, 3 of these patients fulfilled the 2010 revised McDonald criteria for MS (Polman et al., 2011) while the other 15 patients had a CIS. Twenty-one CIS/RRMS-NON patients from the same study were diagnosed as CIS (n = 16) or early RRMS (n = 5) according to the 2010 revised McDonald criteria and had a history of only one neurological attack distinct from ON (e.g. myelitis). Additionally, 23 patients meeting the international consensus diagnostic criteria for NMOSD (Wingerchuk et al., 2015) (19 Aquaporin-4-antibody-positive: 82.6%) (Jarius et al., 2014; Metz et al., 2016; Zekeridou and Lennon, 2015) with a clinically definitive episode of at least one ON (NMOSD-ON) were included from at the time of analysis 53 patients of our neuromyelitis optica observational study (EA1/041/14). NMOSD-ON patients had a time lapse from last ON of 73.2 ± 87.1 months (range: 5–404 months). We enrolled 26 HCs from our imaging research database. Patients were excluded if they 1) were outside age range of 18–70, 2) suffered from ophthalmological defects other than ON, 3) had a history of neurological diseases distinct from MS or NMOSD, 4) had no available DTI acquisition. Further exclusion criteria were similar to general exclusion criteria valid for MRI at 3 T. Part of NMOSD-ON patients' and HCs' DTI data have been investigated and published in a previous study (Oertel et al., 2017). All participants provided written informed consent prior to their inclusion in the study. The study was approved by the local ethics committee and was performed in accordance with the 1964 Declaration of Helsinki in its currently applicable version.

### 2.2 MRI acquisition and analysis

All MRI data were acquired on the same 3 T scanner (Tim Trio Siemens, Erlangen, Germany) using a single-shot echo planar imaging DTI sequence (TR/TE = 7500/86 ms; FOV = 240 × 240 mm^2^; matrix 96 × 96, 61 slices no gap, slice thickness 2.3 mm, 64 non-colinear directions, b-value = 1000 s/mm^2^), a volumetric high-resolution T1 weighted magnetization prepared rapid acquisition gradient echo (MPRAGE) sequence (TR/TE/TI = 1900/2.55/900 ms, FOV = 240 × 240 mm^2^, matrix 240 × 240, 176 slices, slice thickness 1 mm) as well as a volumetric high-resolution fluid-attenuated inversion recovery sequence (3D FLAIR) (TR/TE/TI = 6000/388/2100 ms; FOV = 256 × 256 mm^2^, slice thickness 1.0 mm). 3D FLAIR images of all patients were checked and verified for total lesion volume and OR-specific lesion volume by three expert raters under the supervision of a board-certified radiologist. Whole-brain segmentation and quantification of lesions of FLAIR images were performed using ITK-SNAP (www.itksnap.org) (Yushkevich et al., 2006).

### 2.3 Image processing

#### 2.3.1 Tract-based spatial statistics analysis (TBSS)

DTI data analysis was carried out using TBSS (Smith et al., 2006) with tools from the FMRIB Software Library (FSL 5.0.9).

**Fig. S1:**
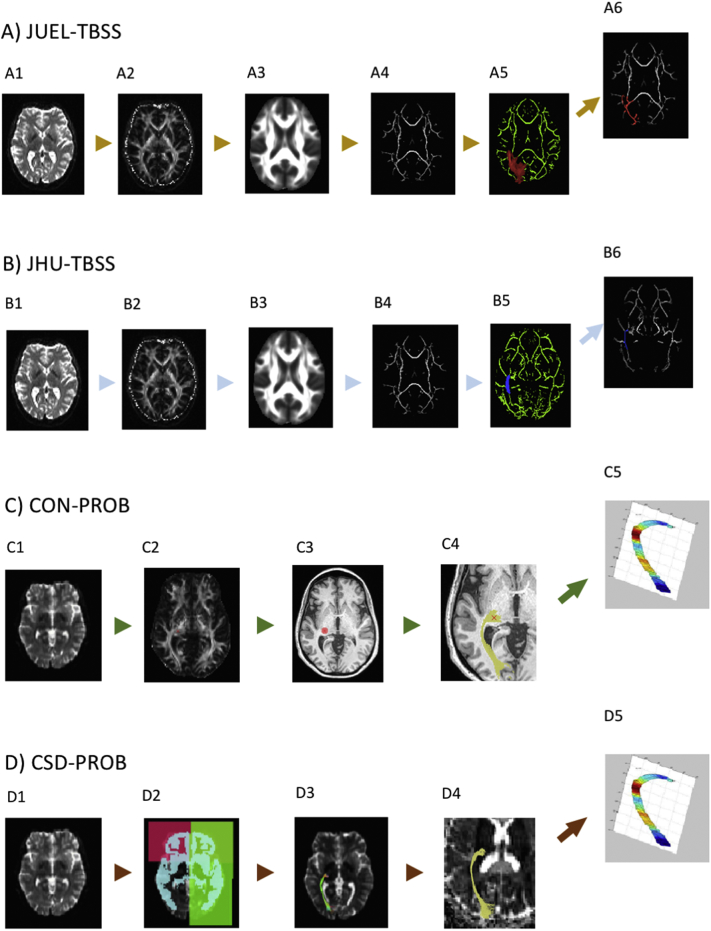
Image processing pipelines. **A** and **B**: JUEL-TBSS and JHU-TBSS - Using (A1 and B1) raw DW imaging data, (A2 and B2) FA maps were created by fitting a tensor model. After (A3 and B3) brain-extraction a mean FA image was created and thinned to produce a mean FA skeleton. (A4 and B4) Each subject's aligned FA data was then projected onto this skeleton. Either (A5) Juelich probabilistic atlas ROI with FA skeleton mask was applied (JUEL-TBSS) and (A6) thresholded, excluding the lower 10%, or (B5) JHU posterior thalamic radiation atlas ROI combined with FA skeleton mask was applied on each subject's aligned FA data (B6) to generate the mean FA. **C**: CON-PROB - (C1) Raw DW imaging data were used to calculate (C2) FA maps within vistalab environment. (C3) LGN ROI was placed manually and (C4) optic radiation was calculated using Contrack algorithm. (C5) Resulting fibers were used to compute tract profiling diffusion properties. **D**: CSD-PROB - Maps of fiber orientation distribution (D1) were calculated using CSD from DW image. (D2) Juelich atlas based LGN and V1 ROI were used as seed mask and target masks. Additionally, sagittal, coronal and grey matter exclusion ROIs were registered from MNI152 to registered from atlas to individual DWI space. (D3) A set of 10.000 streamlines was generated and a threshold of 25% of the maximum value was applied. Resulting fibers were (D4) transferred to Vistalab environment to (D5) compute tract profiling diffusion properties.

First, eddy-current and motion correction were run in FSL, then FA images were created by fitting a tensor model to the raw diffusion data using a least-squares algorithm in FDT, and then brain-extracted using BET (Smith, 2002). FA data were then aligned into a common space using the nonlinear registration tool FNIRT which uses a b-spline representation of the registration warp field. Next, the mean FA image was created and thinned to produce a mean FA skeleton that represents the centres of all tracts common to the group. Each subject's aligned FA data was then projected onto this skeleton (Supplemental Fig. S1A and B; see Supplemental material for further method description).

TBSS skeleton masks were overlaid with two different atlas masks: (A) OR ROIs derived from the Juelich 1 mm probabilistic atlas optic radiation ROI thresholded to exclude the lowest 10% (JUEL-TBSS) and (B) Johns Hopkins University 1 mm white matter tractography probabilistic atlas' posterior thalamic radiation ROI (JHU-TBSS) (Hua et al., 2008; Wakana et al., 2007).

#### 2.3.2 ConTrack probabilistic tractography and Vistalab tract profiling

DTI data analysis was performed using the open-source mrVista package (http://vistalab.stanford.edu/software). Probabilistic fiber tracking was performed using the Contrack algorithm (CON-PROB) (Sherbondy et al., 2008a, Sherbondy et al., 2008b), designed to identify the most likely pathway between two ROIs. Prior to tractography, Eddy current-induced distortion correction and motion correction were performed in all subjects within the vistalab framework. The schematic diagram is shown in Supplemental Fig. S1C (see also Supplemental material for pipeline details). Fiber tensors were fitted using a least-squares algorithm. The eigenvalue decomposition of the diffusion tensors was computed and FA measures were derived along the OR bundles, at 50 equally-spaced positions, resulting in an FA tract profile (Raz et al., 2015). Measurements were calculated by taking a weighted average of the measurements of each individual fiber at the node (so called “fiber core”) (Yeatman et al., 2012) to combine measures throughout the length of the fibers across different subjects.

#### 2.3.3 CSD-based probabilistic tractography and Vistalab tract profiling

We applied a combination of previously published OR tractography based on high order fiber orientation distributions estimated with CSD (CSD-PROB) (Lim et al., 2015; Martínez-Heras et al., 2015) and weighted mean diffusivity calculation as well as tract profiling performance in Vistalab (Yeatman et al., 2012). Probabilistic tractography from seed to target masks was performed in each hemisphere using the MRtrix3 package (http://www.mrtrix.org/) (Tournier et al., 2004, Tournier et al., 2007, Tournier et al., 2008). First, diffusion image preprocessing was performed, including eddy current-induced distortion correction and inter-volume subject motion correction by the use of MRtrix3-in-built usage of FSL's eddy tool (Andersson and Sotiropoulos, 2016; Smith et al., 2004).

Maps of the fiber orientation distributions (FODs) were calculated using CSD with a maximum harmonic order of 6 (CSD algorithm). OR reconstruction pipeline was modified after Martínez-Heras et al. and Lim et al. (Lim et al., 2015; Martínez-Heras et al., 2015) with non-linear transformation of atlas ROIs in MNI space to individual T1 space using FSL FNIRT (Smith et al., 2004) and subsequent registration of ROIs from individual T1 space to individual DWI space using FSL FLIRT (Jenkinson et al., 2002). The tensors were fitted using a linear least squares approach. The schematic diagram of the pipeline is presented in Supplemental Fig. S1D (see also Supplemental material for pipeline details). We then used the resulting fibers to transfer them into the Vistalab environment and compute tract profiling and weighted mean FA of each tract modified after the procedure outlined in the CON-PROB and Vistalab profiling section.

### 2.4 Optical coherence tomography and visual acuity assessment

Optical coherence tomography (OCT) investigations were performed in all CIS/RRMS-NON patients, in 17 out of 18 CIS/RRMS-ON patients, in 22 out of 23 NMOSD-ON patients and in 21 out of 26 HC using a Heidelberg Engineering Spectralis spectral domain OCT (Heidelberg Engineering, Heidelberg, Germany) with automatic real-time (ART) function for image averaging. The peripapillary retinal nerve fiber layer (pRNFL) was measured with activated eye tracker using 3.4-mm ring scans around the optic nerve head (12°, 1536 A-scans 16 ≤ ART ≤ 100). Segmentation of global RNFL was performed semiautomatically using software provided by the OCT manufacturer (Eye Explorer 1.9.10.0 with viewing module 6.0.9.0; Heidelberg Engineering). Visual acuity tests were performed by either using ETDRS charts or the Traditional Snellen Eye Chart in all CIS/RRMS-NON, in 17 out of 18 CIS/RRMS-ON patients, in 21 out of 23 NMOSD-ON patients and in 21 out of 26 HC. Visual testing outcomes were converted in decimals.

### 2.5 Statistical analysis

For statistical analysis we used Graphpad Prism 6.0 (Graphpad Software, San Diego, CA, USA) software and R version 3.1.2 with packages psych, geepack, irr, ICC, lme4, ROCt and ggplot. For comparison and correlation of absolute FA values between methods we used separate FA values of left and right OR and conducted a two-way repeated measures ANOVA to account for the effect of 1) method choice and 2) OR side on FA values within each patient group and an intraclass correlation coefficient (ICC) analysis. Agreement of FA values between methods was evaluated by Pearson's correlation coefficient analysis and Bland-Altman plots (BA-analysis within Graphpad Prism 6.0).

Exploratory comparisons of patient groups regarding T2 lesion volume, RNFL and visual acuity of worse eye were conducted using one-way ANOVA. For group comparisons and correlation analyses with clinical data, we combined FA measures of left and right optic radiation and calculated the simple mean of both values in JHU-TBSS, JUEL-TBSS and CON-PROB. Since OR volumes differed between right and left side in CSD-PROB, we used weighted mean of both values for CSD-PROB based group comparison and correlation analyses. Comparisons of patient groups regarding FA values were assessed using linear model analyses to account for FA values with subsequent R^2^ effect size measures estimation. A receiver operating characteristic (ROC) analysis was used to assess sensitivity and specificity of methods to discriminate each patient group from healthy controls corrected for age. Comparison of tract profiles was conduected using two-way ANOVA comparing FA values of patient groups in every node against HC group. Correction for multiple comparison was performed using Bonferroni correction. Correlations between OR FA values and OR T2 lesion volume, RNFL and visual acuity were performed using linear model analysis. For all statistical analyses, a p-value of <0.05 was regarded as significant. Data are presented as mean ± SD, except for tract profling results that are displayed in mean ± standard error of the mean (SEM).

## 3 Results

### 3.1 Method comparison

#### 3.1.1 Image processing quality

All four methods successfully generated visually appropriate OR tracts, with the exception of one subject in the CIS/RRMS-ON group using the CSD-PROB method.

#### 3.1.2 Coefficient of variation in healthy controls

Coefficient of variation in HC group was lowest in JUEL-TBSS (3.99%) and highest in CON-PROB (13.54%) with comparable coefficients of variation in JHU-TBSS (5.88%) and CSD-PROB (7.21%).

**Table 2:** ICC analysis results of method comparisons by patient group.

	All patients	HC	CIS/RRMS-NON	CIS/RRMS-ON	NMOSD-ON
All methods^a^	0.155⁎	0.024	0.208⁎	0.252⁎	0.074⁎
JUEL-TBSS vs. JHU-TBSS	0.389	0.215	0.391	0.350	0.527
JUEL-TBSS vs. CON-PROB	0.129⁎	−0.084	0.236⁎	0.300⁎	−0.006
JUEL-TBSS vs. CSD-PROB	0.140	0.048	0.175	0.162	0.100
JHU-TBSS vs. CON-PROB	0.122	0.004	0.205	0.260	−0.003
JHU-TBSS vs. CSD-PROB	0.432⁎	0.232⁎	0.447⁎	0.578⁎	0.246⁎
CON-PROB vs. CSD-PROB	0.165	−0.014	0.085	0.155	0.061

HC = healthy control; CIS/RRMS-NON = clinically isolated syndrome without prior optic neuritis; CIS/RRMS-ON = clinically isolated syndrome with prior optic neuritis; NMOSD-ON = neuromyelitis optica spectrum disorder with prior optic neuritis; JUEL-TBSS = Juelich histological atlas optic radiation ROI based tract-based spatial statistics; JHU-TBSS = Johns Hopkins University atlas posterior thalamic ROI based tract-based spatial statistics; CON-PROB = Contrack-based probabilistic tractography; CSD-PROB = constrained spherical deconvolution based probabilistic tractography.

⁎ p < 0.05.

a ICC analysis of all 4 methods (JUEL-TBSS, JHU-TBSS, CON-PROB and CSD-PROB).

**Table 3:** Pearson correlation analysis between all methods by patient groups.

	All patients		HC		CIS/RRMS-NON		CIS/RRMS-ON		NMOSD-ON	
	Pearson's *r*	p-Value	Pearson's *r*	p-Value	Pearson's *r*	p-Value	Pearson's *r*	p-Value	Pearson's *r*	p-Value
JUEL-TBSS vs. JHU-TBSS	0.8714	<0.0001⁎	0.8076	<0.0001⁎	0.8523	<0.0001⁎	0.8683	<0.0001⁎	0.9272	<0.0001⁎
JUEL-TBSS vs. CON-PROB	0.2730	0.0003⁎	−0.2188	0.1191	0.4967	0.0008⁎	0.6002	0.0001⁎	−0.0151	0.9207
JUEL-TBSS vs. CSD-PROB	0.4186	<0.0001⁎	0.2707	0.0523	0.4543	0.0025⁎	0.6094	0.0002⁎	0.2614	0.0864
JHU-TBSS vs. CON-PROB	0.3508	<0.0001⁎	0.0164	0.9084	0.4494	0.0028⁎	0.7063	<0.0001⁎	−0.0119	0.9372
JHU-TBSS vs. CSD-PROB	0.4883	<0.0001⁎	0.2940	0.0344⁎	0.5134	0.0005⁎	0.6428	<0.0001⁎	0.2836	0.0621
CON-PROB vs. CSD-PROB	0.2270	0.003⁎	−0.0629	0.6576	0.1509	0.3398	0.5423	0.0013⁎	0.2695	0.0769

HC = healthy control; CIS/RRMS-NON = clinically isolated syndrome without prior optic neuritis; CIS/RRMS-ON = clinically isolated syndrome with prior optic neuritis; NMOSD-ON = neuromyelitis optica spectrum disorder with prior optic neuritis; JUEL-TBSS = Juelich histological atlas optic radiation ROI based tract-based spatial statistics; JHU-TBSS = Johns Hopkins University atlas posterior thalamic ROI based tract-based spatial statistics; CON-PROB = Contrack-based probabilistic tractography; CSD-PROB = constrained spherical deconvolution based probabilistic tractography.

⁎ p < 0.05.

**Table 4:** Bias and limits of agreement of Bland-Altman analysis.

	Estimation of bias		95% Limits of agreement (LOA)	
	Bias	SD of bias	From	To
JUEL-TBSS vs. JHU-TBSS	0.0466	0.0206	0.0063	0.0871
JUEL-TBSS vs. CON-PROB	0.0218	0.0728	−0.1209	0.1646
JUEL-TBSS vs. CSD-PROB	−0.0664	0.0452	−0.1550	0.0221
JHU-TBSS vs. CON-PROB	0.0922	0.0612	−0.0279	0.2123
JHU-TBSS vs. CSD-PROB	−0.0198	0.0453	−0.1085	0.0689
CSD-PROB vs. CON-PROB	0.1121	0.0708	−0.0266	0.2508

SD = standard deviation; JUEL-TBSS = Juelich histological atlas optic radiation ROI based tract-based spatial statistics; JHU-TBSS = Johns Hopkins University atlas posterior thalamic ROI based tract-based spatial statistics; CON-PROB = Contrack-based probabilistic tractography; CSD-PROB = constrained spherical deconvolution based probabilistic tractography.

**Fig. 2:**
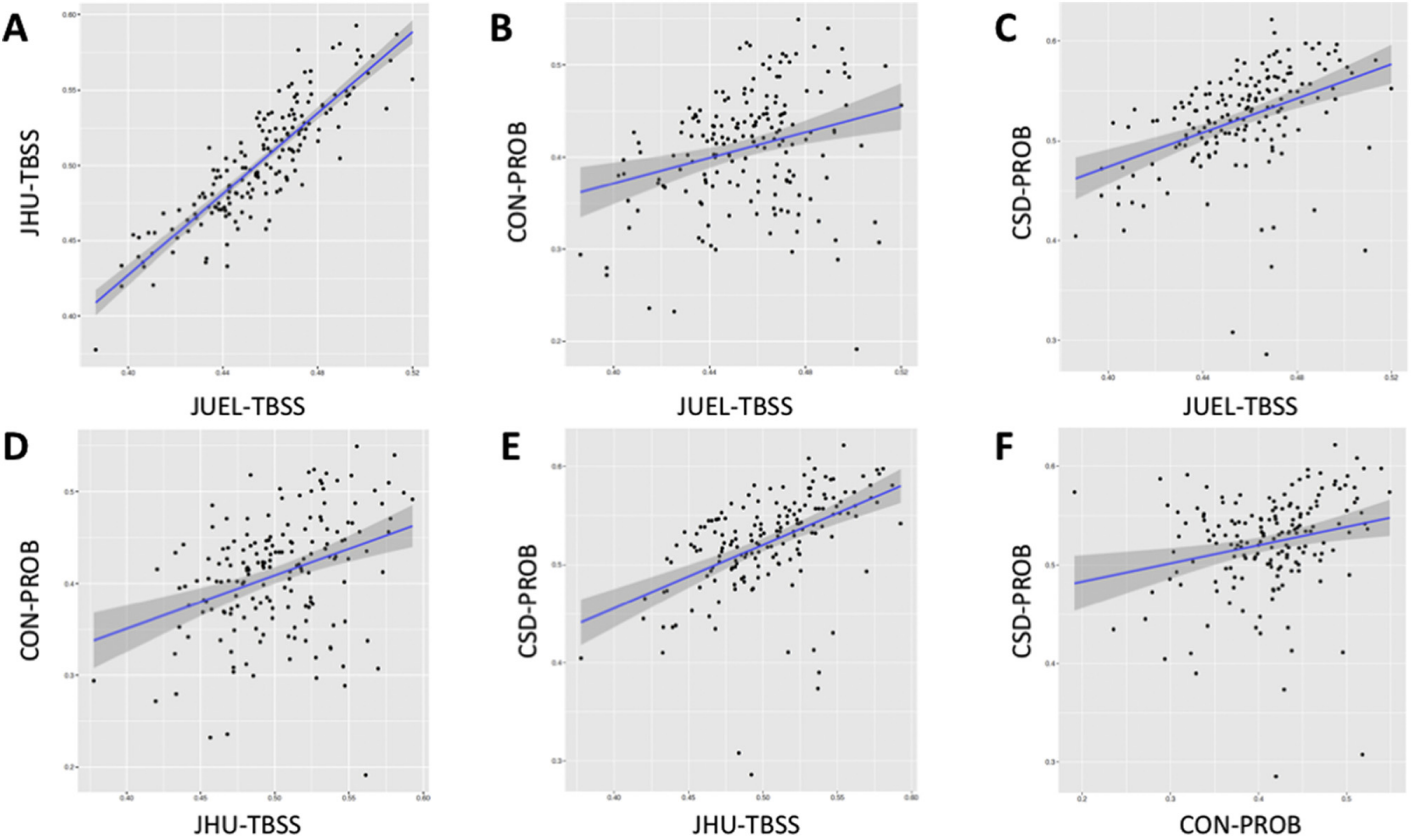
Correlation of all FA values regarding each method. Correlation of all OR FA values assessing **A** JUEL-TBSS vs. JHU-TBSS, **B** JUEL-TBSS vs. CON-PROB, **C** JUEL-TBSS vs. CSD-PROB, **D** JHU-TBSS vs. CON-PROB, **E** JHU-TBSS vs. CSD-PROB, **F** CON-PROB vs. CSD-PROB. JUEL-TBSS = Juelich-based atlas ROI TBSS approach; JHU-TBSS = Johns-Hopkins University posterior thalamic radiation ROI TBSS approach; CON-PROB = ConTrack-based probabilistic tractography. CSD-PROB = constrained spherical deconvolution based probabilistic tractography. TBSS = tract-based spatial statistics; OR = optic radiation.

**Fig. 1:**
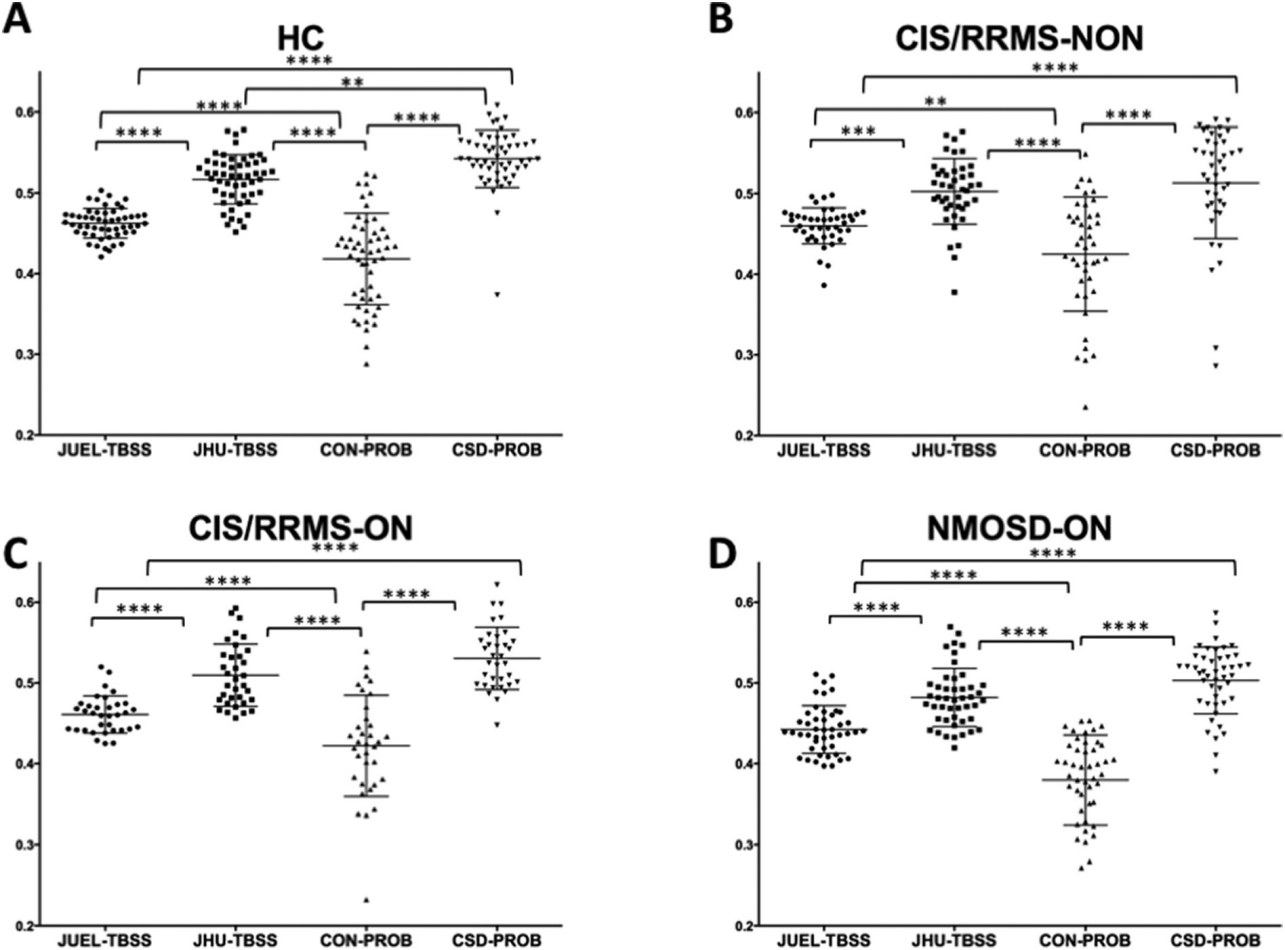
Absolute FA values of different DTI post-processing methods. Optic radiation FA values are shown for **A** healthy controls (HC), **B** CIS patients without prior optic neuritis, **C** CIS patients with optic neuritis in their medical history and **D** NMOSD-ON patients. Comparison of FA distribution yielded significant differences between all methods except for the comparison of JHU-TBSS and CSD-PROB in CIS/RRMS-NON, CIS/RRMS-ON and NMOSD-ON patients. JUEL-TBSS = Juelich-based atlas ROI TBSS approach; JHU-TBSS = Johns-Hopkins University posterior thalamic radiation ROI TBSS approach; CON-PROB = ConTrack-based probabilistic tractography. CSD-PROB = constrained spherical deconvolution based probabilistic tractography. TBSS = tract-based spatial statistics.

#### 3.1.3 Comparison of FA values between methods

**Fig. S2:**
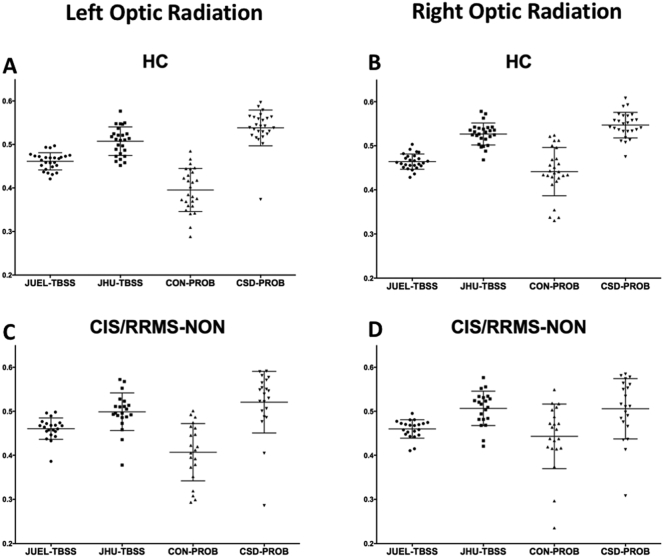

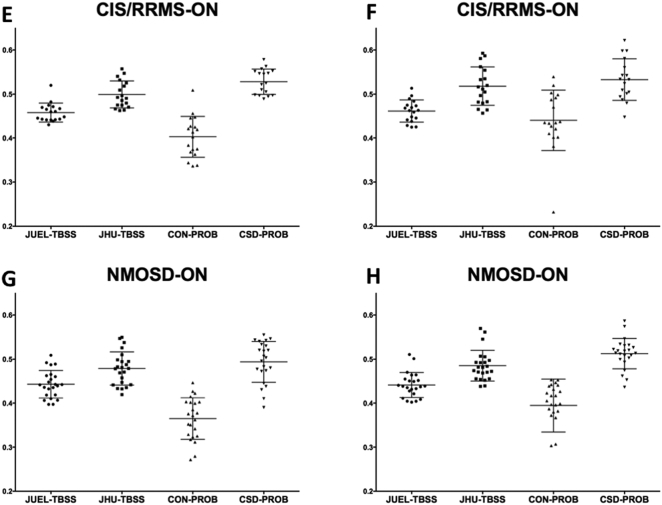
Absolute FA values of different DTI post-processing methods within each subject group. FA values of left and right optic radiation are shown for separately for Optic radiation FA values are shown for **A** and **B** healthy controls (HC), **C** and **D** CIS/RRMS patients without prior optic neuritis, **E** and **F** CIS/RRMS patients with optic neuritis in their medical history and **G** and **H** NMOSD-ON patients. JUEL-TBSS = Juelich-based atlas ROI TBSS approach; JHU-TBSS = Johns-Hopkins University posterior thalamic radiation ROI TBSS approach; CON-PROB = ConTrack-based probabilistic tractography. CSD-PROB = constrained spherical deconvolution based probabilistic tractography. TBSS = tract-based spatial statistics.

Absolute FA value distribution of the different methods for ORs of both sides within each subject group are shown in Fig. 1 (separate left and right OR FA values are shown in Fig. S2). Two-way repeated measures ANOVA revealed a significant impact of 1) method choice and 2) OR side on FA values (see supplementary material, Table S1). Post-hoc tests with Bonferroni correction revealed significant differences between all methods except for the comparison of JHU-TBSS and CSD-PROB in CIS/RRMS-NON, CIS/RRMS-ON and NMOSD-ON. ICC analysis of absolute agreement of all FA values between methods showed poor agreement between methods with ICC values ranging from 0.112 to 0.432 (see Table 2). Lower ICC values were found in patient groups with no suspected visual system damage (HC) and in NMOSD-ON with highest suspected OR damage whereas higher ICC agreement was found in patient groups with suspected moderate damage (CIS/RRMS-ON and CIS/RRMS-NON-group).

#### 3.1.4 Inter-method agreement of FA values

**Fig. S3:**
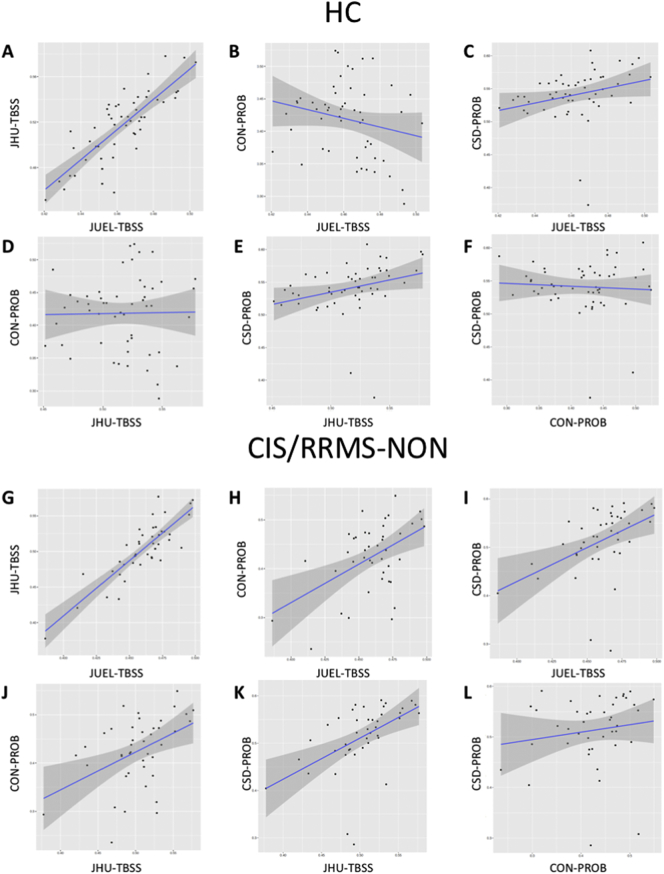

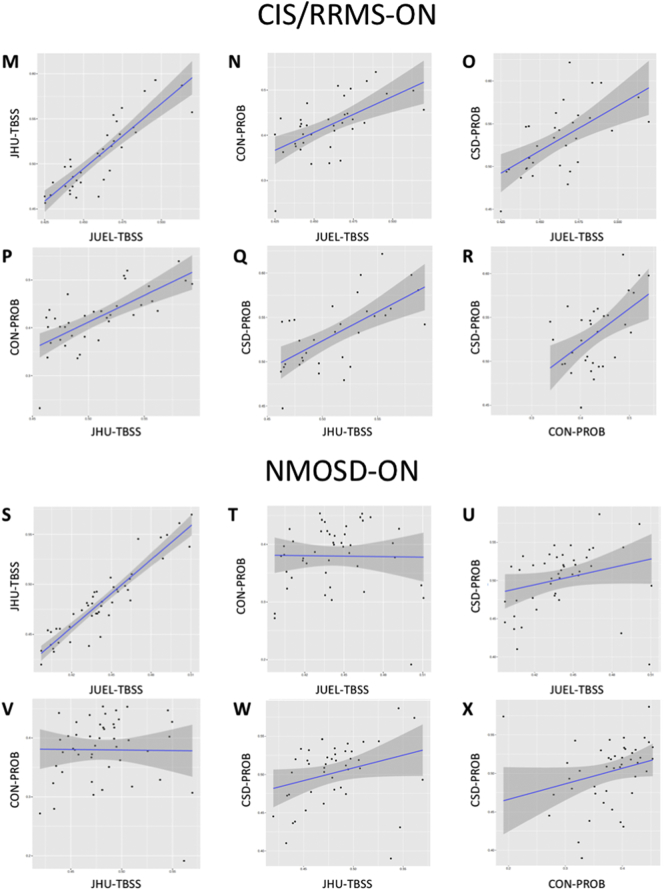
Correlation of FA values of each method by subject group. Correlation of OR FA values of every method (JUEL-TBSS vs. JHU-TBSS; JUEL-TBSS vs. CON-PROB; JUEL-TBSS vs. CSD-PROB; JHU-TBSS vs. CON-PROB; JHU-TBSS vs. CSD-PROB; CON-PROB vs. CSD-PROB) by subject groups HC (**A**–**F**), CIS/RRMS-NON (**G**–**L**), CIS/RRMS-ON (**M**–**R**), and NMOSD-ON (**S**–**X**). JUEL-TBSS = Juelich-based atlas ROI TBSS approach; JHU-TBSS = Johns-Hopkins University posterior thalamic radiation ROI TBSS approach; CON-PROB = ConTrack-based probabilistic tractography. CSD-PROB = constrained spherical deconvolution based probabilistic tractography. TBSS = tract-based spatial statistics; OR = optic radiation; HC = healthy controls; CIS/RRMS-NON = CIS patients with no prior optic neuritis; CIS/RRMS-ON = CIS patients with prior optic neuritis; NMOSD-ON = Neuromyelitis optica spectrum disorder patients with prior optic neuritis.

Pearson correlation analysis revealed significant correlations when analyzing all methods against each other with Pearson's *r* ranging from 0.2730 (JUEL-TBSS vs. CON-PROB) to 0.8714 (JUEL-TBSS vs. JHU-TBSS; see Table 3, Fig. 2, Fig. S3).

**Fig. S4:**
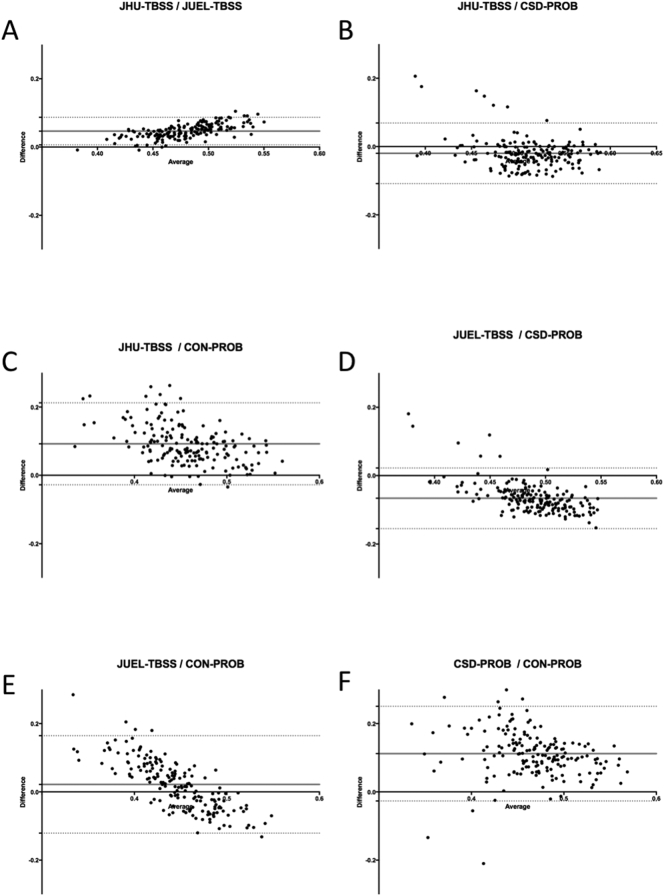
Bland-Altman analysis of mean FA values comparing all methods. Bland-Altman analysis of individual FA values from all subjects including both left and right OR. Middle lines indicate mean differences and dashed lines are limits of agreement. **A** JUEL-TBSS and JHU-TBSS OR ROI masking; **B** TBSS-JHU and CSD-PROB; **C** JHU-TBSS and CON-PROB; **D** JUEL-TBSS and CSD-PROB; **E** JUEL-TBSS and CON-PROB; **F** CSD-PROB and CON-PROB. JUEL-TBSS = Juelich-based atlas ROI TBSS approach; JHU-TBSS = Johns-Hopkins University posterior thalamic radiation ROI TBSS approach; CON-PROB = ConTrack-based probabilistic tractography. CSD-PROB = constrained spherical deconvolution based probabilistic tractography. TBSS = tract-based spatial statistics; OR = optic radiation.

Bland-Altman plots showed good agreement between all methods with most FA values ranging within the 95% confidence interval from average of differences. Best limits of agreement (LOA) were observed between values of both TBSS-based approaches (LOA distance: 0.0934; see Supplemental Fig. S4A; Table 4). Comparison of probabilistic tractography based methods with TBSS based methods generally showed a proportional error with overestimation of high FA values and underestimation of low FA values in probabilistic tractography (Fig. S4C and E). Best agreement of relative and absolute FA values between all methods were seen at medium FA values (0.45–0.5) suggesting good agreement in methods in identifying minimal to medium damage.

### 3.2 Comparison of patient groups against healthy controls

#### 3.2.1 Patient group differences from HC and effect size

**Fig. 3:**
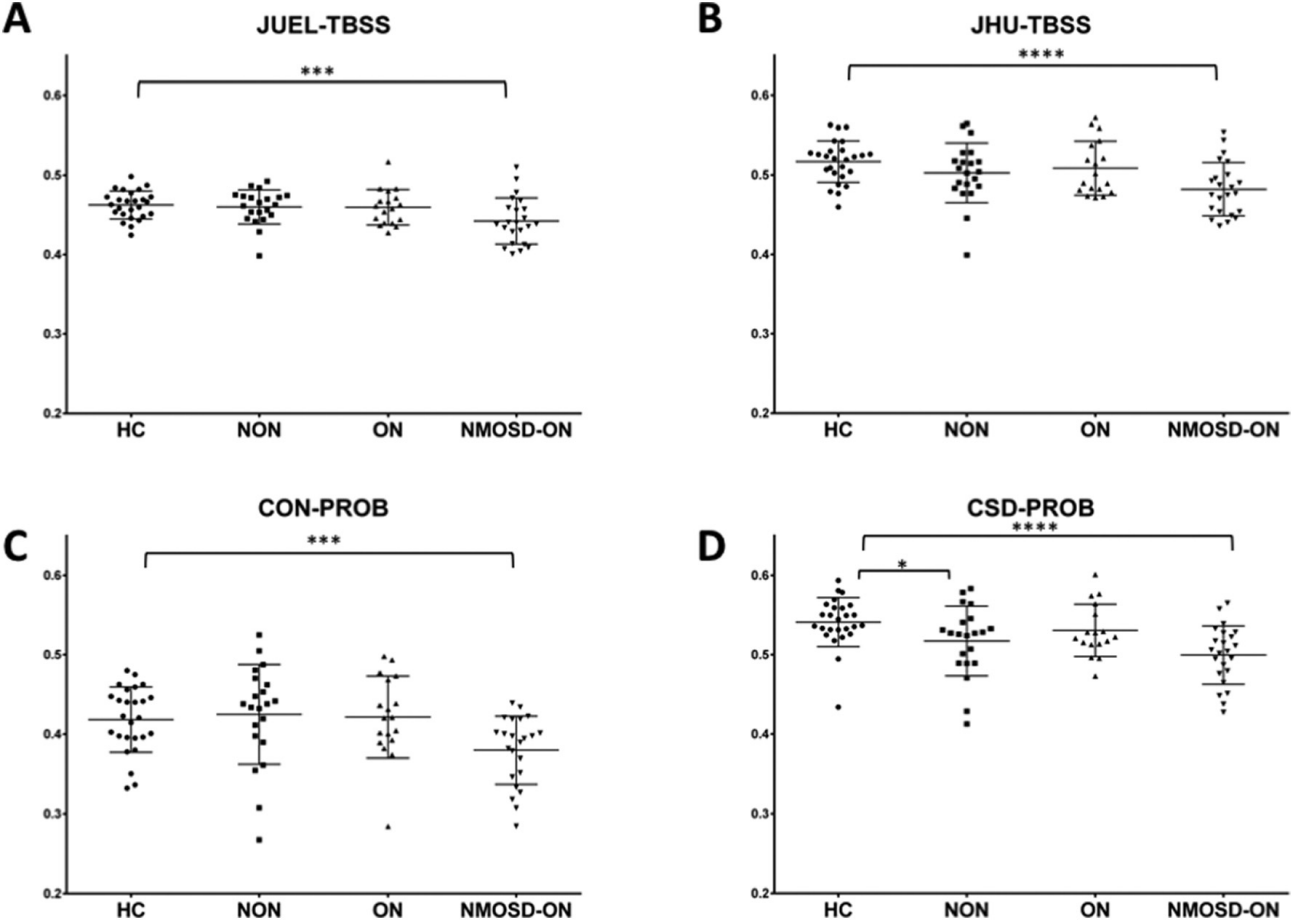
OR mean FA comparison of patient groups and HCs arranged by methods. Mean FA distribution of individual TBSS skeletons within JUEL-TBSS (**A**) and JHU-TBSS (**B**). Both approaches show significant differences between HC and NMOSD group. JHU also shows significant differences between NMOSD and all CIS groups and differences between HC and CIS and HC and CIS/RRMS-NON. Comparison of weighted mean FA distribution within CON-PROB tracts (**C**) and CSD-PROB OR fibers (**D**) reveal similar significant differences between HC and NMOSD and NMOSD with all CIS/RRMS-subgroups. CSD-PROB also reveals significant differences between HC and all CIS-subgroups. FA = fractional anisotropy; HC = healthy controls; OR = optic radiation; TBSS = tract-based spatial statistics; JHU = Johns Hopkins University; ROI = region of interest; CSD = constrained spherical deconvolution; NMOSD = neuromyelitis optica spectrum disorder. * p < 0.05; ** p < 0.005; *** p < 0.0005; **** p < 0.0001.

Comparison of patient groups against healthy controls regarding T2 lesion volume, OCT RNFL, visual acuity and optic radiation FA values are shown in Table 5. All patient groups showed higher T2 lesion volume and increased OR specific T2 lesion volume compared to HC. RNFL was significantly decreased compared to HC in CIS/RRMS-ON and NMOSD-ON, while visual impairment was only seen in NMOSD-ON group. Linear model analysis of FA differences between each patient group and healthy controls showed FA differences between NMOSD-ON and HCs throughout all methodologies (JUEL-TBSS: p = 0.0043; JHU-TBSS: p = 0.0002; CON-PROB: p = 0.0024; CSD-PROB: p < 0.0001; Fig. 3). p-Values and R^2^ as the effect size and proportion of variance explained by the method are displayed in Table 6. Highest effect size in the discrimination of HC and NMOSD was seen in CSD-PROB (R^2^ = 0.282). CSD-PROB revealed significant FA differences between CIS/RRMS-ON patients and HCs, that were not observable when other methods were applied.

**Table 5:** Comparison of patient groups against healthy controls regarding T2 lesion volume, visual parameters and optic radiation FA values.

	HC	CIS/RRMS-NON	CIS/RRMS-ON	NMOSD-ON	ANOVA p
Total T2 lesion volume [ml; mean ± sd]	0.38 ± 0.66	2.87⁎ ± 4.39	2.59⁎ ± 3.17	2.15⁎ ± 3.07	0.084
OR-specific T2 lesion volume [ml; mean ± sd]	0.04 ± 0.07	0.70⁎ ± 1.02	0.57⁎ ± 0.70	0.44⁎ ± 0.87	0.017⁎
RNFL [μm; mean ± sd]	96.90 ± 7.50	98.21 ± 12.16	87.92⁎ ± 14.76	67.12⁎ ± 19.72	<0.001⁎
Visual acuity of worse eye [mean ± sd]	1.02 ± 0.31	1.00 ± 0.37	0.96 ± 0.29	0.74⁎ ± 0.47	0.003⁎
FA [JUEL-TBSS]	0.46 ± 0.02	0.46 ± 0.02	0.46 ± 0.02	0.44⁎ ± 0.03	0.012⁎
FA [JHU-TBSS]	0.52 ± 0.03	0.50 ± 0.04	0.51 ± 0.04	0.48⁎ ± 0.04	0.004⁎
FA [CON-PROB]	0.42 ± 0.06	0.43 ± 0.07	0.42 ± 0.06	0.38⁎ ± 0.06	0.010⁎
FA [CSD-PROB]	0.54 ± 0.03	0.52⁎ ± 0.04	0.53 ± 0.03	0.50⁎ ± 0.04	0.001⁎

HC = healthy control; CIS/RRMS-NON = clinically isolated syndrome without prior optic neuritis; CIS/RRMS-ON = clinically isolated syndrome with prior optic neuritis; NMOSD-ON = neuromyelitis optica spectrum disorder with prior optic neuritis; FA = fractional anisotropy; JUEL-TBSS = Juelich histological atlas optic radiation ROI based tract-based spatial statistics; JHU-TBSS = Johns Hopkins University atlas posterior thalamic ROI based tract-based spatial statistics; CON-PROB = Contrack-based probabilistic tractography; CSD-PROB = constrained spherical deconvolution based probabilistic tractography.

Exploratory ANOVA and subsequent *t*-test p-values.

⁎ p < 0.05 (significant from HC).

**Table 6:** FA differences between patient groups and healthy controls.

	CIS/RRMS-NON vs. HC		CIS/RRMS-ON vs. HC		NMOSD-ON vs. HC	
	p-Value	R^2^	p-Value	R^2^	p-Value	R^2^
JUEL-TBSS	0.661	0.004	0.628	0.005	0.004⁎	0.161
JHU-TBSS	0.134	0.049	0.362	0.020	<0.001⁎	0.262
CON-PROB	0.663	0.004	0.818	0.001	0.002⁎	0.179
CSD-PROB	0.035⁎	0.094	0.298	0.026	<0.001⁎	0.282

HC = healthy control; CIS/RRMS-NON = clinically isolated syndrome without prior optic neuritis; CIS/RRMS-ON = clinically isolated syndrome with prior optic neuritis; NMOSD-ON = neuromyelitis optica spectrum disorder with prior optic neuritis; JUEL-TBSS = Juelich histological atlas optic radiation ROI based tract-based spatial statistics; JHU-TBSS = Johns Hopkins University atlas posterior thalamic ROI based tract-based spatial statistics; CON-PROB = Contrack-based probabilistic tractography; CSD-PROB = constrained spherical deconvolution based probabilistic tractography.

⁎ p < 0.05.

#### 3.2.2 ROC-analysis

**Fig. 4:**
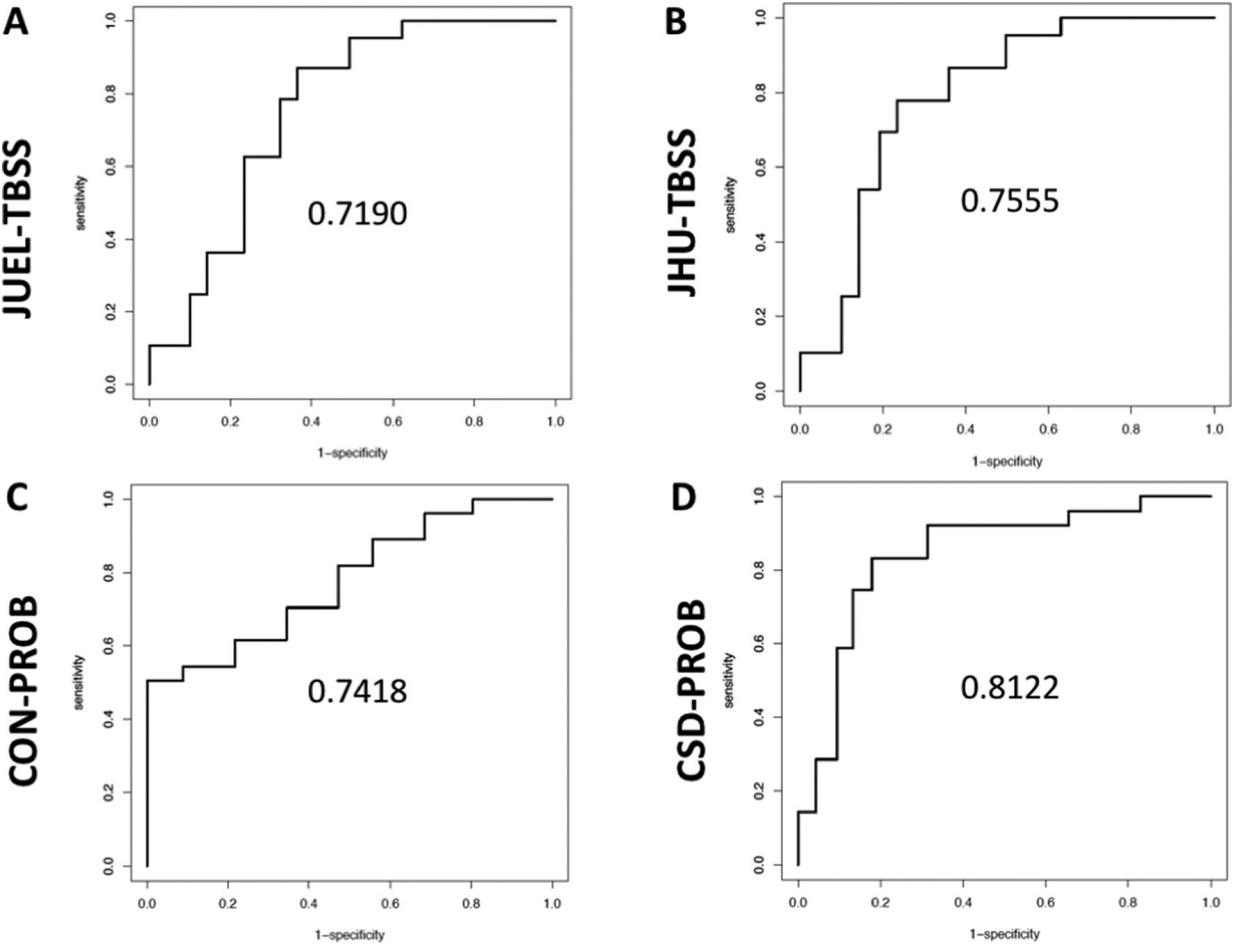
ROC curves and AUCs for TBSS and CSD-based analysis methods. ROC curves and AUCs are displayed comparing HC with NMOSD corrected for age by use of **A** JUEL-TBSS, **B** JHU-TBSS, **C** CON-PROB and **D** CSD-PROB. ROC = receiver operating characteristics; AUC = area under the curve; HC = healthy controls; NMOSD = neuromyelitis optica spectrum disorder; TBSS = tract-based spatial statistics; CSD = constrained spherical deconvolution; JHU = Johns Hopkins University.

AUC values to discriminate HCs from NMOSD-ON were highest in CSD-PROB (AUC = 0.812), while slightly lower in CON-PROB (AUC = 0.742), JHU-TBSS (AUC = 0.756) and JUEL-TBSS (AUC = 0.719; Fig. 4). ROC-analysis results of comparison between HC vs. CIS/RRMS-ON and HC vs. CIS/RRMS-NON are shown in Table 7.

**Table 7:** AUC values for the comparison of patient groups against healthy controls by each method corrected for age.

Method	Group 1	Group 2	AUC
JUEL-TBSS	HC	CIS/RRMS-NON	0.611
JUEL-TBSS	HC	CIS/RRMS-ON	0.625
JUEL-TBSS	HC	NMOSD-ON	0.719
JHU-TBSS	HC	CIS/RRMS-NON	0.743
JHU-TBSS	HC	CIS/RRMS-ON	0.704
JHU-TBSS	HC	NMOSD-ON	0.756
CON-PROB	HC	CIS/RRMS-NON	0.704
CON-PROB	HC	CIS/RRMS-ON	0.523
CON-PROB	HC	NMOSD-ON	0.742
CSD-PROB	HC	CIS/RRMS-NON	0.796
CSD-PROB	HC	CIS/RRMS-ON	0.626
CSD-PROB	HC	NMOSD-ON	0.812

HC = healthy control; CIS/RRMS-NON = clinically isolated syndrome without prior optic neuritis; CIS/RRMS-ON = clinically isolated syndrome with prior optic neuritis; NMOSD-ON = neuromyelitis optica spectrum disorder with prior optic neuritis; JUEL-TBSS = Juelich histological atlas optic radiation ROI based tract-based spatial statistics; JHU-TBSS = Johns Hopkins University atlas posterior thalamic ROI based tract-based spatial statistics; CON-PROB = Contrack-based probabilistic tractography; CSD-PROB = constrained spherical deconvolution based probabilistic tractography.

#### 3.2.3 Tract profiling – subject group comparison

**Fig. 5:**
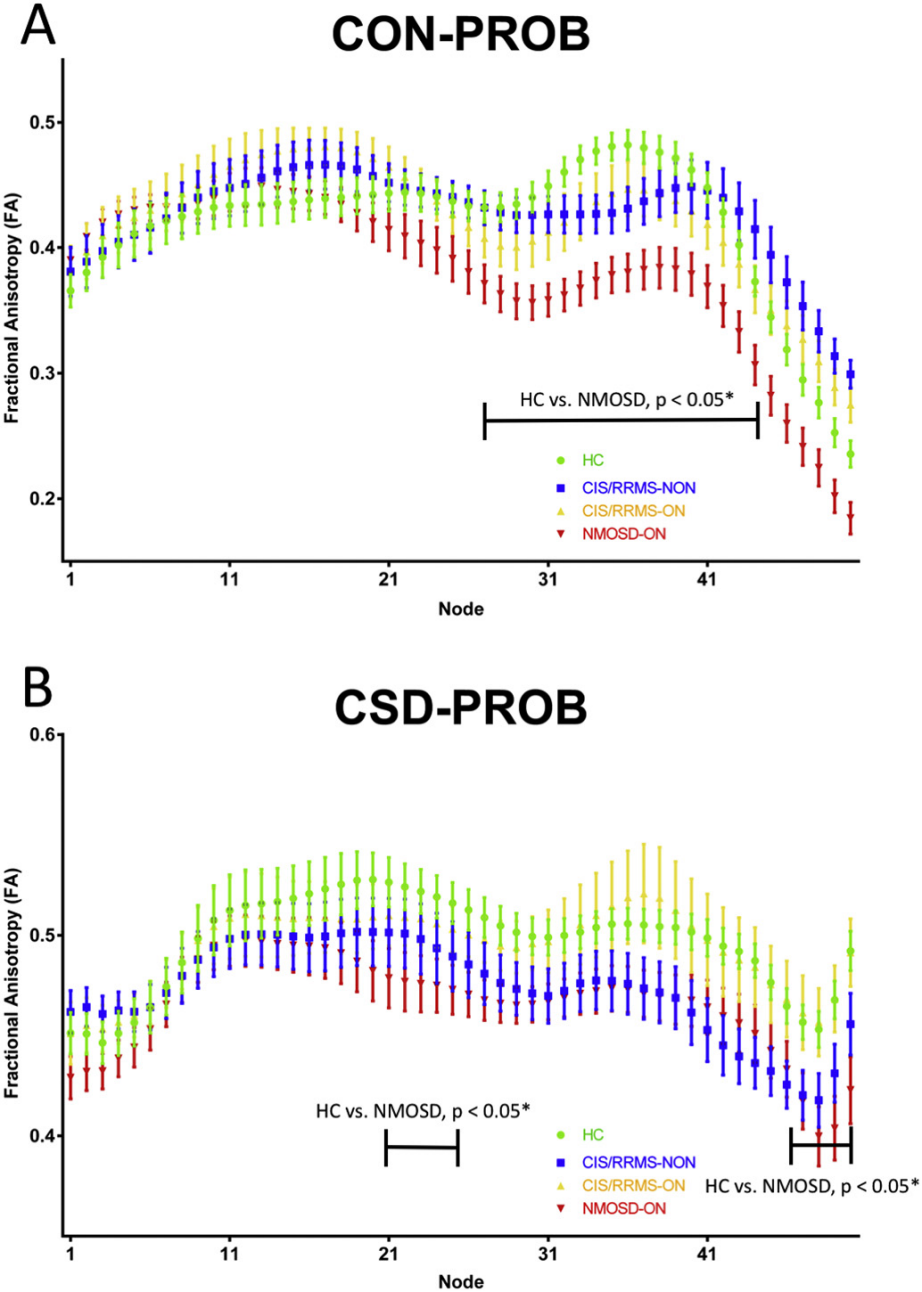
Tract profiles of the optic radiation in different patient groups. OR partitioning into 50 equally divided nodes in NMOSD (red), CIS/RRMS-ON (orange) and CIS/RRMS-NON (yellow) patients and Healthy controls (green) using (**A**) Contrack-based probabilistic tractography (**B**) CSD-based tractography. OR = optic radiation; CIS = clinically isolated syndrome; ON = optic neuritis; CSD = constrained spherical deconvolution; NMOSD = neuromyelitis optica spectrum disorder; FA = fractional anisotropy.

Tract profiles comparing patient groups are shown in Fig. 5. Significant differences between NMOSD-were seen in both methods (CON-PROB: nodes 26–47; CSD-PROB: nodes 20–25 and 48–50; Fig. 5).

#### 3.2.4 OR-specific lesions and OR FA

We investigated possible correlations between OR FA values and optic radiation specific lesion volume. JUEL-TBSS, JHU-TBSS and CSD-PROB showed significant correlations between FA and OR T2 lesion volume in the CIS/RRMS-NON group (see Table 8).

**Table 8:** Correlations of optic radiation specific lesion volume, RNFL and visual acuity with optic radiation FA values by method.

	JUEL-TBSS			JHU-TBSS			CON-PROB			CSD-PROB		
	Estimate	Std error	p-Value	Estimate	Std error	p-Value	Estimate	Std error	p-Value	Estimate	Std error	p-Value
OR T2 Lesion volume												
HC	−0.073	0.060	0.23	−0.124	0.089	0.17	0.152	0.142	0.29	0.026	0.108	0.24
CIS/RRMS-NON	−0.022	0.005	**<0.01**⁎	−0.044	0.008	**<0.01**⁎	−0.022	0.017	0.21	−0.031	0.012	**0.01**⁎
CIS/RRMS-ON	−0.011	0.009	0.26	−0.022	0.015	0.15	−0.014	0.023	0.54	−0.027	0.014	0.06
NMOSD-ON	−0.010	0.007	0.18	−0.013	0.008	0.15	−0.016	0.011	0.15	−0.003	0.009	0.76

RNFL												
HC	0.001	0.000	0.57	0.001	0.000	0.98	0.001	0.000	0.37	0.001	0.000	0.21
CIS/RRMS-NON	0.001	0.000	**<0.01**⁎	0.002	0.000	**<0.01**⁎	0.002	0.000	**0.01**⁎	0.001	0.001	**0.01**⁎
CIS/RRMS-ON	0.001	0.000	0.06	0.001	0.000	0.24	0.001	0.000	0.10	0.001	0.000	0.07
NMOSD-ON	0.000	0.000	0.52	0.001	0.000	0.74	0.001	0.000	0.32	0.001	0.000	0.44

Visual acuity												
HC	0.022	0.008	**0.01**⁎	0.019	0.019	0.12	−0.055	0.019	**<0.01**⁎	0.024	0.011	**0.03**⁎
CIS/RRMS-NON	0.026	0.008	**<0.01**⁎	0.041	0.015	**0.01**⁎	0.021	0.027	0.44	0.010	0.017	0.55
CIS/RRMS-ON	0.012	0.011	0.30	0.014	0.017	0.45	0.033	0.024	0.17	0.011	0.019	0.56
NMOSD-ON	0.016	0.001	0.10	0.012	0.011	0.28	0.006	0.014	0.68	0.003	0.012	0.77

JUEL-TBSS = Juelich histological atlas optic radiation ROI based tract-based spatial statistics; JHU-TBSS = Johns Hopkins University atlas posterior thalamic ROI based tract-based spatial statistics; CON-PROB = Contrack-based probabilistic tractography; CSD-PROB = constrained spherical deconvolution based probabilistic tractography. RNFL = retinal nerve fiber layer thickness. Significant p-values are displayed in bold.

⁎ p < 0.05.

#### 3.2.5 RNFL and OR FA

Associations between RNFL and OR FA were exclusively shown in the CIS/RRMS-NON group by all methods (see Table 8).

#### 3.2.6 Visualy acuity and OR FA

Associations between visual acuity and OR FA were exclusively shown in the CIS/RRMS-NON group by JUEL-TBSS and JHU-TBSS. (see Table 8).

## 4 Discussion

Our study compared TBSS and probabilistic tractography based approaches to quantify OR damage in patients with NMOSD-ON and CIS with and without ON. While the distribution of absolute FA values differed among methods, correlation analyses and Bland-Altman plots revealed good agreement of FA values, especially in FA magnitudes of suspected mild OR damage, reflected by OR-specific lesion load and RNFL decrease (CIS/RRMS-ON and CIS/RRMS-NON). Both, TBSS and probabilistic tractography methods detected microstructural damage in NMOSD-ON patients compared to HCs.

### 4.1 Robustness of methods

CSD-PROB failed to generate OR tracts in one CIS/RRMS-ON patient, while successfully generating tracts in all other subjects. All other methods successfully identified the ORs in all subjects. It has been reported, that extensive white matter lesions in neurological disorders, such as stroke or multiple sclerosis, may lead to erroneous termination of the tracking algorithm or may cause a deviation of the bundles at the level of the lesions (Ciccarelli et al., 2008). A previous study in stroke patients showed that a CSD-based approach resulted in successful corticospinal tract reconstruction in 76 out of 78 tracts, while a comparative DTI-based fiber tractography resulted in the corticospinal tract reconstruction in 67 out of 78 potential tracts (Auriat et al., 2015). For both approaches unsuccessful fiber tract reconstruction occurred in the ipsilesional hemisphere of participants, indicating lesions to be responsible for insufficient tract generation and different tractography to yield distinct susceptibilities towards lesion-associated tract generation failure (Auriat et al., 2015). In our study, unsatisfactory tract generation in our CIS/RRMS-ON patient using CSD-PROB might be caused by extensive white matter lesions that were observed in the patient's optic radiations.

### 4.2 Inter-method comparison of FA distribution

In our study, CON-PROB showed highest coefficient of variation of FA in HC, while JUEL-TBSS showed lowest coefficient of variation. Supposing that a homogeneous and normally distributed cohort was investigated, low coefficients of variation may suggest a correlate of good method quality. High coefficients of variation in HCs in CON-PROB, possibly caused by the mainly manual approach, might impair the validity of the method. However, high coefficients may on the other hand indicate higher method sensitivity. A recently published study compared a) individual CON-PROB with b) healthy control-based CON-PROB template OR reconstructions and c) Juelich histological atlas-based OR ROI approach in 35 healthy controls and 70 MS patients (Wang et al., 2018). Despite differences in the reconstructed OR volumes, both OR lesion volume and OR diffusivity measurements in MS subjects were highly comparable in this study. The authors found diffusivity differences between different OR segmentation techniques to be consistently small across low and high values.

By contrast, the distribution of absolute OR FA values significantly differed in our study between nearly all methods in all patient groups and showed poor absolute agreement in the ICC analysis, except for JHU-TBSS and CSD-PROB. We conclude that differences between absolute OR FA values may impede comparisons of previous and future DTI study results investigating microstructural OR damage. The application of the exact same method is therefore necessary to allow for any statements on possible differences between OR FA values within a specific cohort of patients. These findings may be of particular significance in any case of OR DTI comparison, regardless of within-study analyses or comparisons of OR DTI results between studies, for example in meta-analyses. Comparisons of absolute OR DTI values that did not use the same post-processing approach are not valid and must therefore be avoided.

### 4.3 Inter-method comparison of FA correlations and agreement

OR FA values of all methods showed significant correlations suggesting underlying associations of FA values and actual OR specific microstructural damage regardless of method choice. Subgroup analyses of Pearson correlation coefficient analyses revealed best correlations of OR FA values in CIS/RRMS-ON and CIS/RRMS-NON. These findings are in line with a recent study reporting on good agreement between CON-PROB, template-based OR reconstruction and a Juelich OR ROI-based approach in HC and MS measured by Pearson correlation coefficents and Bland-Altman analysis (Wang et al., 2018).

By contrast, only limited correlations of OR FA values were seen in our study in the non-damage group (HC) and patients with suspected extensive OR damage (NMOSD-ON).

In a recent study, CSD-PROB was investigated in ten HCs and five MS patients to compare tractography results with histological masks. It showed a good sensitivity ranging from 65% to 81% and a specificity up to 100% (Martínez-Heras et al., 2015). Another recent study compared CSD-PROB with Juelich histological atlas in 20 patients with various neurological conditions, showing a good match of the probabilistic tractography approach with a mean AUC of 0.87 (Lim et al., 2015). These findings are in line with our study showing relatively little bias between JUEL-TBSS masking approach and CSD-PROB in the conducted Bland-Altman analysis. Bland-Altman analysis revealed best agreement between all methods at medium FA values (0.45–0.5) suggesting good agreement of methods in identifying damage of medium magnitude (CIS/RRMS-ON and CIS/RRMS-NON). These findings might – at least to a certain extent – suggest the convertibility of results by different DTI post-processing methods when applied to patient groups with OR damage of mild to moderate magnitude. Concrete research approaches to patient groups with suspected mild OR damage, for example CIS patients, could be sufficiently tackled by all investigated methods, while investigations regarding HCs or severely affected patient groups (NMOSD-ON) might lead to different results, highly dependent of the chosen method.

The presence of systematic bias and proportional errors in the comparison of DTI TBSS-based and tractography based methods may lead to false positive or false negative results when different patient groups are compared by different methods. While one method might produce significant differences in group comparison due to underestimation of low FA values, another method may yield non-significant results due to relative overestimation of low FA values. These findings might be a causative factor of today's equivocal findings (Assaf and Pasternak, 2008) of previous DTI visual pathway analyses that impede the evaluation of DTI as a potential biomarker (Inglese and Bester, 2010).

### 4.4 NMOSD vs. HC group comparison

Group comparison showed FA differences between NMOSD-ON and HCs throughout all TBSS and probabilistic tractography based methods. Best effect size and AUC values to distinguish both groups were observed for CSD-PROB. JHU-TBSS, JUEL-TBSS and CON-PROB showed slightly lower AUC values and effect size. Our study results are in line with previous investigations using DTI reporting on microstructural degradation with significant FA reduction within the OR (Oertel et al., 2017; Pache et al., 2016a, Pache et al., 2016b; Rueda Lopes et al., 2012; Yu et al., 2006). A previous study in NMOSD patients, using TBSS, found FA values to be exclusively reduced in regions associated to the visual system by making use of a TBSS ROI and a multivariate comparison approach. These results provide evidence of anterograde trans-synaptic degeneration due to ON (Pache et al., 2016a, Pache et al., 2016b). By contrast, one TBSS-based study demonstrated reduced FA involving not only the OR but also diffuse subcortical white matter structures in frontal, parietal, temporal, occipital and limbic regions (von Glehn et al., 2014). Another study used CSD-PROB OR tractography and revealed FA reductions within the OR of 25 AQP4-antibody seropositive NMOSD patients (Oertel et al., 2017). Notably, OR FA was not only reduced in NMOSD patients with previous ON but FA reductions were also detectable in 6 NMOSD patients with longitudinally extensive transverse myelitis (LETM) without evidence of prior ON. These results were corroborated by another study that used FSL-based probabilistic tractography (FSL's probtrackx) to delineate the OR and found FA reduction within the OR of 24 NMOSD patients with prior ON (58.3% AQP4-antibody seropositive) as well as in 12 NMOSD patients without prior ON (66.6% AQP4-antibody positive) (Tian et al., 2017). These findings suggest microstructural changes in the afferent visual system independent of ON attack-related mechanisms.

Although clinical history of our NMOSD patients with prior unilateral or bilateral ON, long disease duration and pronounced visual impairment and OCT RNFL thinning suggests the presence of attack-related optic radiation FA decrease in our NMOSD cohort, we did not find any direct associations between OCT RNFL or visual acuity and optic radation FA, irrespective of the method. However, our data mirror the clinical experience as well as findings from conventional imaging studies showing that neurological disability and tissue damage in the visual pathway are on average more pronounced in NMOSD as compared to MS/CIS, as it can be seen in the relatively frequent bilateral manifestation of optic neuritis in our NMOSD-cohort compared to CIS/RRMS-ON (Bennett et al., 2015; Kim et al., 2015; Schmidt et al., 2017).

### 4.5 CIS/RRMS-ON and CIS/RRMS-NON vs. HC

No difference of OR FA between CIS/RRMS-ON and HC was seen in any of the methods used. CSD-PROB showed differences between HC and CIS/RRMS-NON. In CIS and early stages of MS, OR microstructural damage is most likely caused by 1) trans-synaptic neurodegeneration after ON (Gabilondo et al., 2014) and 2) impact of T2 inflammatory lesions within the OR (Graham and Klistorner, 2017; Sinnecker et al., 2015a, Sinnecker et al., 2015b).

Damage in the OR of ON patients due to inflammatory T2 lesions has been investigated previously. Raz et al. reported on reduced OR FA by making use of CON-PROB in patients with clinically isolated ON compared to healthy controls. In this study, reduced OR FA was associated with OR specific T2 lesion volume suggesting FA differences to be explained by intrabundle lesions (Raz et al., 2015). In our CIS/RRMS-NON cohort, OR FA in JUEL-TBSS, JHU-TBSS and CSD-PROB was associated with OR specific T2 lesion volume, indicating TBSS approaches to be more sensitive to lesional damage than probabilistic tractography approaches. These findings indicate lesional damage to be at least partly responsible for damage within the ORs of CIS/RRMS patients without prior ON. However, recent findings indicated the presence of a measurable, time-dependent trans-synaptic neurodegeneration effect on the OR after ON, independent of T2 lesion load. Longitudinal investigations using an atlas-based OR template ROI in 38 acute ON patients over 12 months showed FA reduction at baseline and subsequent additional FA decrease at an average rate of −2.6% per year (Kolbe et al., 2016). Another study investigated twenty-eight acute ON patients by use of the FSL based probabilistic tractography algorithm and found no difference between patients' and controls' mean OR FA at baseline but a constant decrease over time after 3, 6 and 12 months (Tur et al., 2016). No associations between RNFL and OR FA were found in CIS/RRMS-ON patients. Given the relatively short time after ON in our CIS/RRMS-ON cohort with a mean disease duration of 4.63 ± 5.15 months after ON, we presume that the early timepoint of MRI acquisition after ON makes the determination of any trans-synaptic effect on the optic radiations unlikely.

### 4.6 Tract profiling using probabilistic tractography methods

Tract-profiling group differences between HC and NMOSD were seen in higher proportion of nodes in CON-PROB compared to CSD-PROB, indicating CON-PROB tract-profiling to yield higher sensitivity for the detection of microstructural OR damage in NMOSD compared to CSD-PROB tract profiling. Using CON-PROB, tract-profiling enabled the distinction between OR fibers affected by T2 lesions and non-lesional OR fibers. Radial diffusivity, mean diffusivity and FA changes were detected along the entire OR, while axial diffusivity changes were confined to the posterior half of the OR. This discrepancy implied distinct pathophysiologic processes to be detectable by DTI tract profiling (Klistorner et al., 2015).

In our study, tract profiling showed middle and posterior parts of the OR to be more affected than anterior OR sections in NMOSD compared to HC. These findings may suggest distinct regions of the OR to exhibit more pronounced damage by trans-synaptic neurodegeneration or distinct OR T2 lesional damage affecting only specific regions of the OR due to Wallerian degeneration (Klistorner et al., 2015). However, in our analysis overall NMOSD OR FA was not associated with optic radiation T2 lesion volume. Distinct regions of the OR are supposed to be less affected by contamination from craniocaudally oriented crossing fibers to the optic radiation. Neighbouring and crossing white matter pathways may additionally lead to a reduced FA in OR fiber regions (Kamali et al., 2014). Exclusive microstructural OR damage is more likely to be observable by DTI in regions that are not affected by crossing or kissing fibers, which are represented by distinct middle and posterior parts of the OR. Both, the affection of the OR by crossing and kissing fibers, as well as distinct damage patterns caused by the localization of OR-specific T2 lesions or trans-synaptic neurodegeneration damage patterns may therefore be the cause of different levels in FA decrease along OR regions.

### 4.7 Technical aspects

TBSS can be implemented fully-automated requiring no manual intervention. In TBSS, the average FA may be affected by surrounding structures due to partial voluming (Smith et al., 2006). Probabilistic tractography is more time-consuming due to manual and calculation processes inherent to the specific algorithm (Wang et al., 2018). Tractography algorithms are known to be - at least to a certain extent - susceptible to image artifacts with possible insufficient tract generation (Auriat et al., 2015). However, the CSD-PROB approach used in our study represents a feasible and fully-automated probabilistic tractography method, requiring no manual intervention compared to previously used CON-PROB (Wang et al., 2018).

### 4.8 Limitations

Given the multitude of methods that exist for tractography and the comparison of DWI measures, our study naturally fails to comprehensively include all alternative methods for comparison. Our study is limited by the small sample size of the respective subpopulations mitigating validity of our cross-method comparison.

## 5 Conclusion

To our knowledge, this is the first study to compare multiple probabilistic tractography and TBSS-based approaches to quantify microstructural OR damage in patients with neuroinflammatory visual pathway damage. We proved TBSS-based and probabilistic tractography based DWI processing techniques to be feasible in detecting microstructural damage within the OR. Absolute FA values differed between the methods, preventing comparisons of OR FA analyses of previous and future studies with different post-processing approaches. Correlation and agreement of all methods' FA values were best in patients with suggested mild to moderate OR FA damage (CIS/RRMS patients), indicating methods to be exchangeable – at least to a certain extent – in the analysis of CIS/RRMS patients but not if healthy controls or patients with suspected severe damage (NMOSD-ON) are investigated. Due to systematic bias and proportional errors of FA between methods, the comparison of subject groups by use of different methods leads to different (either false-positive or false-negative) results. Although the pattern of differences between the patient cohorts was similar in our study, CSD-PROB showed significant FA differences between HC and CIS/RRMS-NON patients. Although these CSD-PROB derived differences between the groups could result from the above-mentioned systematic bias, we suggest CSD-PROB to be more sensitive to early neuroinflammatory damage, partially associated with lesions. All methods were successful in differentiating NMOSD-ON patients from HCs. Given that CSD-PROB showed highest AUC and effect size followed by JHU-TBSS, JUEL-TBSS and CON-PROB, CSD-PROB approach might be the method of choice to further investigate differential diagnostic aspects between HC and NMOSD. Tract-profiling differences between HC and NMOSD were more pronounced in CON-PROB, which might be the method of choice for tract profiling assessments. In our study, TBSS-based approaches showed better correlations with OR specific lesions, which could favor them as the method of choice for future studies to investigate the relationship between T2 lesions and DTI. Given the lack of a “gold-standard” for non-invasive DW-MRI OR delineation (Kuchling et al., 2017; Lim et al., 2015; Thomas et al., 2014), future studies are required to fully validate the capacity and limitations of different post-processing methods with regards not only to differential diagnosis and T2 lesional impact on DTI, but also concerning longitudinal FA assessment and OR DTI relationships with visual function.

The following are the supplementary data related to this article.

Supplementary material Image 1
