# Supplementary material for: Comparison of probabilistic tractography and tract-based spatial statistics for assessing optic radiation damage in patients with autoimmune inflammatory disorders of the central nervous system
Source: Neuroimage Clin. 2018 May 8;19:538–50. doi: 10.1016/j.nicl.2018.05.004 (PMC6029567; doi:10.1016/j.nicl.2018.05.004)
Supplement: Supplementary file 1 — Supplementary material [file mmc1.docx]

Supplemental material

**Comparison of probabilistic tractography and tract-based spatial statistics for assessing optic radiation damage in patients with autoimmune inflammatory disorders of the central nervous system**

Joseph Kuchling^1,2^*, Yael Backner^3^*, Frederike C Oertel^1^, Noa Raz^3^, Judith Bellmann-Strobl^1,4^, Klemens Ruprecht^2^, Friedemann Paul^1,2,4$^, Netta Levin^3^, Alexander U Brandt^1,5§^, Michael Scheel^1§^

*) Equally contributing first authors

^§^) Equally contributing senior authors

^$^) Correspondence: Friedemann Paul, NeuroCure Clinical Research Center, Charité - Universitätsmedizin Berlin, Charitéplatz 1, 10117 Berlin, Germany, Tel. +49 30 450 539705, Fax +49 30 450 539915, email: friedemann.paul@charite.de

# Methods

## Contrack-based optic radiation tractography pipeline

For the ORs, the lateral geniculate nuclei (LGN) and calcarine sulcus (V1) were anatomically defined as ROIs on T1 maps of each subject. CON-PROB generated 75,000 possible pathways out of which the top 20% (15,000) highest-scored fibers were selected as most likely representative of the OR. A few clearly misidentified fibers were eliminated by visual inspection.

## CSD-based optic radiation tractography pipeline

Binary masks of LGN (thresholded by 11%) and V1 (thresholded by 50%) derived from the Juelich probabilistic atlas were created. Additionally, we applied three binary exclusion masks created within MNI-152 space: 1) a termination coronal plane 20 mm posterior to the temporal pole 2) a midline sagittal exclusion plane and 3) ipsilateral GM regions analogous to previously published anatomical exclusion criteria that did not include the seed and target masks derived from SPM-based grey matter segmentation (33). Seed-, target and exclusion-masks were registered to the individual T1 space using FSL-FNIRT, and were then registered from individual T1 to individual DWI space by FSL-FLIRT. A set of 10,000 streamlines was generated unidirectionally from the LGN mask and was terminated immediately after entering the target V1 mask. Tractography parameters were determined as follows: step size: 0.2 mm, curvature threshold: 0.3 mm and FA threshold: 0.1. To account for misidentified fibers we selected a threshold of 25 % of the maximum value derived from the track density image to mitigate the majority of aberrant fibers and to reduce the occurcence of false positive fibers analogous to Lim et al.(10).

# Results

| **source of variation** | **p-value** |
| --- | --- |
| method | <0.001 |
| method*disease | 0.12 |
| side | <0.001 |
| method*side | 0.085 |
| method*side*disease | 0.7783 |

**Table S1.** Two-way repeated measures ANOVA result

|  | Method | HC | CIS/RRMS-NON | CIS/RRMS-ON | NMOSD-ON |
| --- | --- | --- | --- | --- | --- |
| L E F T | JUEL-TBSS | 0.461 ± 0.020 | 0.460 ± 0.024 | 0.458 ± 0.022 | 0.443 ± 0.031 |
|  | JHU-TBSS | 0.507 ± 0.033 | 0.499 ± 0.042 | 0.499 ± 0.031 | 0.479 ± 0.038 |
|  | CON-PROB | 0.395 ± 0.049 | 0.407 ± 0.065 | 0.403 ± 0.038 | 0.365 ± 0.047 |
|  | CSD-PROB | 0.541 ± 0.031 | 0.521 ± 0.045 | 0.522 ± 0.033 | 0.493 ± 0.039 |
| R I G H T | JUEL-TBSS | 0.464 ± 0.017 | 0.460 ± 0.021 | 0.461 ± 0.025 | 0.441 ± 0.028 |
|  | JHU-TBSS | 0.527 ± 0.025 | 0.507 ± 0.039 | 0.518 ± 0.043 | 0.485 ± 0.035 |
|  | CON-PROB | 0.441 ± 0.055 | 0.443 ± 0.073 | 0.440 ± 0.069 | 0.394 ± 0.060 |
|  | CSD-PROB | 0.542 ± 0.026 | 0.513 ± 0.047 | 0.519 ± 0.037 | 0.491 ± 0.030 |

**Table S2.** Mean FA values with SD measured within left and right optic radiations by distinct methods within subject groups.

LEFT=left optic radiation values; RIGHT=right optic radiation values; HC=healthy controls; CIS/RRMS-NON=clinically isolated syndrome without optic neuritis; CIS/RRMS-ON=clinically isolated syndrome with optic neuritis; NMOSD=neuromyelitis optica spectrum disorder. JUEL-TBSS=TBSS approach with Juelich optic radiation atlas region of interest; JHU-TBSS=TBSS approach with Johns Hopkins University atlas region of interest; CON-PROB=ConTrack-based probabilistic tractography; CSD-PROB=constraind spherical deconvolution-based tractography.

**Fig. S1** Image processing pipelines.

**A** and **B:** JUEL-TBSS and JHU-TBSS - Using (A1 and B1) raw DW imaging data, (A2 and B2) FA maps were created by fitting a tensor model. After (A3 and B3) brain-extraction a mean FA image was created and thinned to produce a mean FA skeleton. (A4 and B4) Each subject’s aligned FA data was then projected onto this skeleton. Either (A5) Juelich probabilistic atlas ROI with FA skeleton mask was applied (JUEL-TBSS) and (A6) thresholded, excluding the lower 10 percent, or (B5) JHU posterior thalamic radiation atlas ROI combined with FA skeleton mask was applied on each subject’s aligned FA data (B6) to generate the mean FA.

**C** CON-PROB - (C1) Raw DW imaging data were used to calculate (C2) FA maps within vistalab environment. (C3) LGN ROI was placed manually and (C4) optic radiation was calculated using Contrack algorithm. (C5) Resulting fibers were used to compute tract profiling diffusion properties.

**D:** CSD-PROB - Maps of fibre orientation distribution (D1) were calculated using CSD from DW image. (D2) Juelich atlas based LGN and V1 ROI were used as seed mask and target masks. Additionally, sagittal, coronal and grey matter exclusion ROIs were registered from MNI152 to registered from atlas to individual DWI space. (D3) A set of 10.000 streamlines was generated and a threshold of 25 % of the maximum value was applied. Resulting fibers were (D4) transferred to Vistalab environment to (D5) compute tract profiling diffusion properties.

## **Figure S2.** Absolute FA of values different DTI post-processing methods within each subject group

FA values of left and right optic radiation are shown for separately for Optic radiation FA values are shown for **A and B** healthy controls (HC), **C and D** CIS patients without prior optic neuritis, **E and F** CIS patients with optic neuritis in their medical history and **G and H** NMOSD-ON patients.

JUEL-TBSS = Juelich-based atlas ROI TBSS approach; JHU-TBSS = Johns-Hopkins University posterior thalamic radiation ROI TBSS approach; CON-PROB = ConTrack-based probabilistic tractography. CSD-PROB = constrained spherical deconvolution based probabilistic tractography. TBSS = tract-based spatial statistics;

**Figure S3.** Correlation of FA values of each method by subject group

Correlation of OR FA values of every method (JUEL-TBSS vs. JHU-TBSS; JUEL-TBSS vs. CON-PROB; JUEL-TBSS vs. CSD-PROB; JHU-TBSS vs. CON-PROB; JHU-TBSS vs. CSD-PROB; CON-PROB vs. CSD-PROB) by subject groups HC **(A-F)**, CIS/RRMS-NON **(G-L)**, CIS/RRMS-ON **(M-R)**, and NMOSD-ON **(S-X).**

## **Figure S4.** Bland-Altman analysis of mean FA values comparing all methods

Bland-Altman analysis of individual FA values from all subjects including both left and right OR. **A** JUEL-TBSS and JHU-TBSS OR ROI masking; **B** TBSS-JHU and CSD-PROB; **C** JHU-TBSS and CON-PROB; **D** JUEL-TBSS and CSD-PROB; **E** JUEL-TBSS and CON-PROB; **F** CSD-PROB and CON-PROB

JUEL-TBSS = Juelich-based atlas ROI TBSS approach; JHU-TBSS = Johns-Hopkins University posterior thalamic radiation ROI TBSS approach; CON-PROB = ConTrack-based probabilistic
